# Molecular functions and clinical impact of thyroid hormone-triggered autophagy in liver-related diseases

**DOI:** 10.1186/s12929-019-0517-x

**Published:** 2019-03-08

**Authors:** Hsiang-Cheng Chi, Chung-Ying Tsai, Ming-Ming Tsai, Chau-Ting Yeh, Kwang-Huei Lin

**Affiliations:** 1 Radiation Biology Research Center, Institute for Radiological Research, Chang Gung University/Chang Gung Memorial Hospital, Linkou Taoyuan, Taiwan; 2 Kidney Research Center and Department of Nephrology, Chang Gung Immunology Consortium, Chang Gung Memorial Hospital, Taoyuan, 333 Taiwan; 3 Department of Nursing, Chang-Gung University of Science and Technology, Taoyuan, Taiwan 333; 4 Department of General Surgery, Chang Gung Memorial Hospital, Chiayi, Taiwan 613; 5 Liver Research Center, Chang Gung Memorial Hospital, Linkou Taoyuan, Taiwan 333; 6 Department of Biochemistry, College of Medicine, Chang-Gung University, 259 Wen-Hwa 1 Road, Taoyuan, 333 Taiwan, Republic of China; 7 Research Center for Chinese Herbal Medicine, College of Human Ecology, Chang Gung University of Science and Technology , Taoyuan, Taiwan

**Keywords:** Thyroid hormone, Thyroid hormone receptor, Autophagy, non-alcoholic fatty liver disease, hepatocellular carcinoma

## Abstract

The liver is controlled by several metabolic hormones, including thyroid hormone, and characteristically displays high lysosomal activity as well as metabolic stress-triggered autophagy, which is stringently regulated by the levels of hormones and metabolites. Hepatic autophagy provides energy through catabolism of glucose, amino acids and free fatty acids for starved cells, facilitating the generation of new macromolecules and maintenance of the quantity and quality of cellular organelles, such as mitochondria. Dysregulation of autophagy and defective mitochondrial homeostasis contribute to hepatocyte injury and liver-related diseases, such as non-alcoholic fatty liver disease (NAFLD) and liver cancer.

Thyroid hormones (TH) mediate several critical physiological processes including organ development, cell differentiation, metabolism and cell growth and maintenance. Accumulating evidence has revealed dysregulation of cellular TH activity as the underlying cause of several liver-related diseases, including alcoholic or non-alcoholic fatty liver disease and liver cancer. Data from epidemiologic, animal and clinical studies collectively support preventive functions of THs in liver-related diseases, highlighting the therapeutic potential of TH analogs. Elucidation of the molecular mechanisms and downstream targets of TH should thus facilitate the development of therapeutic strategies for a number of major public health issues.

Here, we have reviewed recent studies focusing on the involvement of THs in hepatic homeostasis through induction of autophagy and their implications in liver-related diseases. Additionally, the potential underlying molecular pathways and therapeutic applications of THs in NAFLD and HCC are discussed.

## Background

Thyroid hormones (TH) serve as potent regulators of cellular development, growth and metabolism in mammals [1] and control several metabolic activities related to anabolism or catabolism of macromolecules, including carbohydrates, proteins, lipids and damaged organelles within cells to maintain homeostasis under different physiological conditions [2]. In addition to their critical regulatory roles in cellular homeostasis, imbalance of TH levels in the body is associated with multiple chronic diseases, including diabetes mellitus [3, 4], cardiovascular disease [5, 6] and liver-related disorders [7]. Liver is one of the most important target organs whereby THs regulate components involved in cellular metabolism, such as fatty acids, supporting the possibility that disruption of TH action in liver contributes to development of non-alcoholic fatty liver disease (NAFLD). Indeed, recent studies have reported associations between alterations in cellular TH signaling and several liver-related diseases, including NAFLD and hepatocellular carcinoma (HCC). Earlier epidemiological findings suggest that long-term hypothyroidism is positively associated with high risk of NAFLD and HCC incidence, independent of other risk factors [8, 9]. Moreover, treatment with T_3_ or its analogs has been shown to prevent a spectrum of liver-related diseases ranging from hepatic steatosis to HCC in rodents subjected to high-fat diet (HFD) or carcinogens [10–17]. These collective findings support the potential utility of TH analogs as therapeutic drugs to prevent liver disease progression. Analysis of the downstream signals of TH in liver may further shed light on the underlying TH pathways that induce therapeutic effects against liver-related diseases.

Autophagy is a self-digestion process primarily involving recycling of cellular fuel stores in lysosomes to generate amino acids, glucose and fatty acids [18]. Catabolism of lipids through autophagy is termed lipophagy [19]. In addition to metabolic functions, autophagy presents a cellular surveillance mechanism to suppress accumulation of toxic protein aggregates and impairment of organelles, thus facilitating maintenance of organelle integrity and cellular homeostasis [20]. The specific regulatory functions of autophagy in hepatic homeostasis have been increasingly explored in recent years. Dysregulation of the autophagic process is reported to cause an imbalance in energy metabolism in the liver and consequently affect hepatic physiology and trigger disease [21–24]. Several research groups, including ours, have shown that hepatic lipid turnover is stimulated by THs through lipophagy, preventing hepatosteatosis, both in *vitro* and in *vivo* [25, 26]. Additionally, TH stimulates the metabolic rate accompanied by increased mitochondrial turnover through mitophagy, leading to elimination of mitochondrial dysfunction induced by hepatic carcinogens or hepatitis B virus HBx protein [16, 17, 27]. The finding that THs and Thyroid hormone receptors (THRs) prevent hepatic damage, hepatosteatosis and hepatocarcinogenesis via autophagy stimulation supports their therapeutic potential in clinical applications. In the current report, we have reviewed studies published by our research group and other investigators on the involvement of TH-induced autophagy in liver-related diseases, particularly NAFLD and HCC. Elucidation of the network of molecular mechanisms underlying the effects of TH/THR on hepatic metabolism may aid in the design of effective therapeutic strategies for a range of liver-related diseases.

### Molecular actions of thyroid hormones and receptors

#### Genomic actions of TH

T_3_ (triiodothyronine) and T_4_ (L-thyroxine) are the two major thyroid hormones affecting almost every organ system. Under physiological conditions, T_4_ is the main hormone secreted into the bloodstream by the thyroid gland. However, the thyroid hormone receptor (THR) binding affinity of T_4_ is considerably lower (10-fold less) than that for T_3_. The conversion of T_4_ to T_3_ is regulated via iodothyronine deiodinases (DIO1, DIO2, and DIO3) in extrathyroidal tissue. Type I and type II iodothyronine deiodinases (DIO1, DIO2) deiodinate circulating T_4_ to produce biologically active T_3_. Conversely, type III deiodinase (DIO3) suppresses intracellular thyroid activity by converting T_4_ and T_3_ to the comparatively inactive forms, reverse T_3_ (rT_3_) and T_2_. Recently, T_2_ was shown to possess thyromimetic activity and mimic some of the effects of T_3_ on liver metabolism [28, 29], implying that T_2_ or rT_3_ may not just be inert metabolites as originally suggested. Expression levels and activities of DIO1, DIO2 and DIO3 vary among different tissues, causing a tissue-specific increase or decrease in circulating TH levels or availability of active hormones for THR binding [7, 30]. To exert genomic effects, cytoplasmic T_3_ enters the nucleus, most likely through passive diffusion, and binds THRs associated with thyroid hormone response elements (TRE) within the promoter regions of downstream genes of TH/THR [31–33]. Typical TREs within promoter regions of downstream genes contain two half-site sequences (A/G)GGT(C/A/G)A in a palindromic, direct repeat or inverted repeat arrangement that are recognized by THR [1].

THRs are T_3_-inducible transcription factors belonging to the nuclear receptor superfamily that are encoded by two tissue-specific genes, *THRA* (TRα) and *THRB* (TRβ). The *THRA* gene encodes one active T_3_-binding receptor, TRα1, and two dominant-negative spliced variants, TRΔα1 and TRΔα2 [34]. that lack T_3_ binding ability [35]. TRα1 is the predominant subtype highly expressed in brain, cardiac and skeletal muscle [36]. *THRB* encodes two functional T_3_-binding TRβ isoforms (TRβ1 and TRβ2) and another dominant-negative isoform, TRβ4 [34]. TRβ1 is predominately expressed in brain, liver and kidney whereas TRβ2 is limited to the hypothalamus, retina and pituitary. THRs exert transcriptional effects via formation of homodimers or heterodimers with other nuclear receptors, such as retinoid X receptor (RXR), Vitamin D receptors (VDR) and other retinoic acid receptor subtypes. RXR generally functions as a partner of several nuclear receptors to regulate target genes [47]. THRs form heterodimers with RXR on TREs within the promoter regions of target genes. In addition, recent ChIP-Seq studies have shown that THRs bind to specific response element motifs with non-conserved sequences and in non-promoter regions [37–39], implying that interactions with other transcription factors are required to regulate chromatin remodeling and gene expression.

**Fig. 1:**
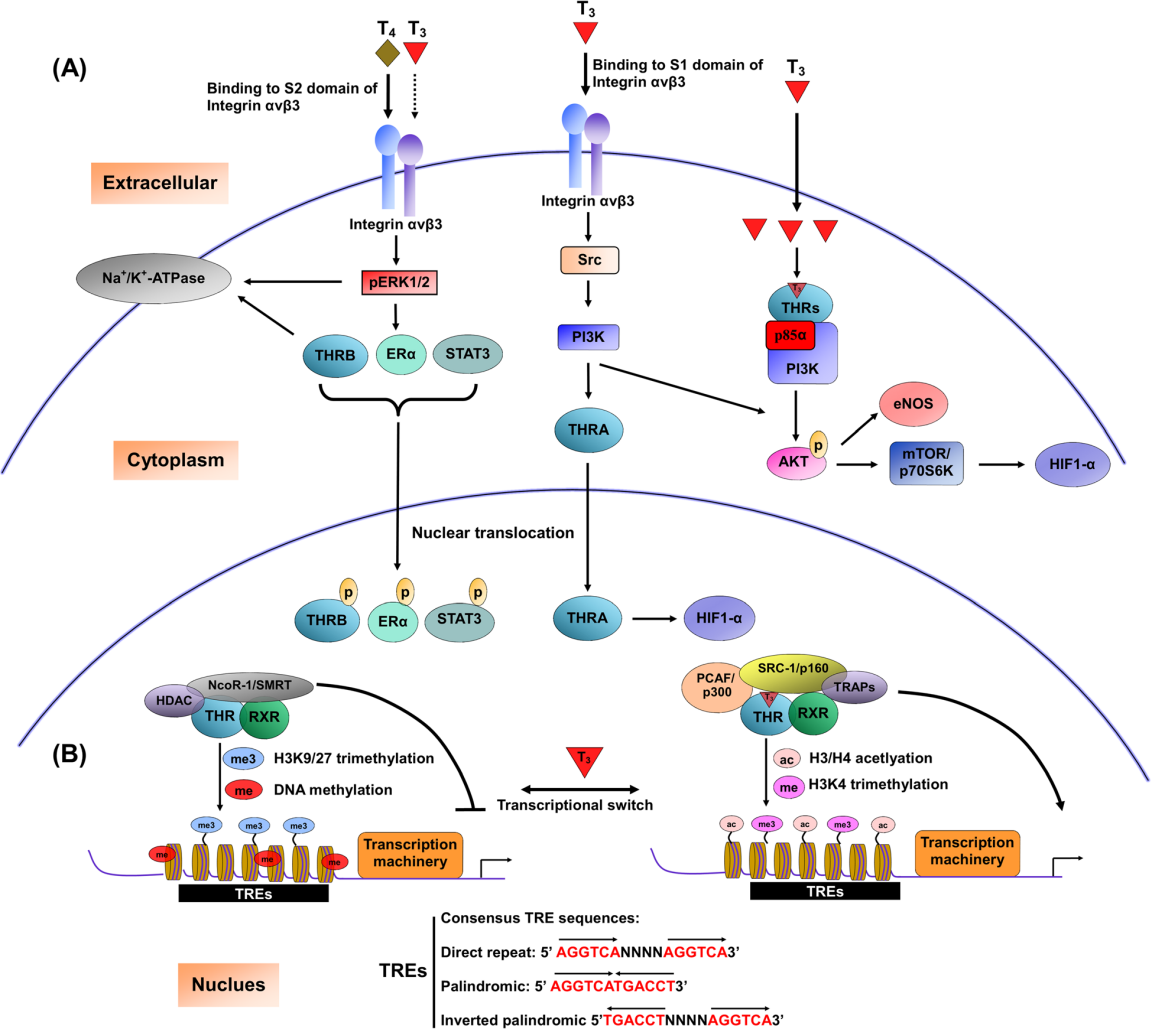
Nongenomic and Genomic actions of Thyroid hormone and thyroid hormone receptor. The diagram of nongenomic and transcriptional actions of thyroid hormone (TH) and thyroid hormone receptor (THR). **a** Nongenomic effects of THs are initiated from Integrin αvβ3 localized on the plasma membrane or occurs at cytoplasm. T3 interacts with S1 domain of Integrin αvβ3 to activate the PI3K signal pathway via Src kinase, leading to trafficking of THRA from the cytoplasm to nucleus and increases HIF-1α expression. THs, mainly T4, also interact with S2 domain of Integrin αvβ3 to activate ERK 1/2 signal, causing phosphorylation and nuclear localization of THRβ, estrogen receptor α (ERα) and STAT3. Activated ERK1/2 and cytosolic THRB increase the activity of the sodium pump (Na, K-ATPase). T3-liganded THRs in the cytoplasm interact with the PI3K regulatory subunit, p85α, to activate Akt, subsequently triggering mTOR/p70S6K and eNOS signals. **b** In the nucleus, THRs form heterodimers with the retinoid X receptor (RXR) at thyroid hormone response elements (TREs), within the regulatory regions of downstream genes. In the absence of T_3_, the co-repressor complex involving histone deacetylase (HDACs), NCoR1 and SMRT deacetylate histones in the regulatory regions. Consequently, trimethylation of histone H3 at lysine 9 and 27 along with DNA methylation causes a more closed conformation in chromatin and blocks the transcriptional machinery access to the DNA, causing suppression of downstream targets transcription. Binding of T3 induces conformational changes of THRs and recruitment of transcriptional coactivators (such as PCAF/P300 and SRC-1/p160) with histone acetyltransferase (HAT) activity to increase histone acetylation at specific promoter regions, facilitating generation of a permissive chromatin state and further recruitment of general transcriptional machinery. Typical TREs within promoter regions of downstream genes contain two half-site sequences (A/G)GGT(C/A/G)A in a palindromic, direct repeat or inverted repeat arrangement that are recognized by THR

In the absence of TH, THRs still bind to TREs but are associated with co-repressors displaying histone deacetylase (HDAC) activity, leading to modifications in chromatin structure and repression of transcription. For instance, nuclear receptor corepressor 1 (NCoR1) and silencing mediator for retinoid or thyroid-hormone receptors (SMRT), well-characterized co-repressors with histone deacetylase activity, serve as platforms for repressor complex-mediated chromatin remodeling [40]. Binding of T_3_ induces conformational changes of THR and recruitment of transcriptional coactivators with histone acetyl transferase (HAT) activity to increase histone acetylation at specific promoter regions, facilitating generation of a permissive chromatin state and further recruitment of general transcriptional machinery (Fig. 1). For instance, steroid hormone receptor coactivator (SRC), PCAF (p300/CBP-associated factor) and p160 family members facilitate ligand-bound THRs to activate T_3_ target genes through histone acetyltransferase activity [41]. Moreover, transcriptional activities of THRs are stimulated by TR-associated protein (TRAP) family independently of HAT activity [42]. Alterations in THR-associated co-regulator complexes may induce differential responses for appropriate target gene expression (Table 1).

**Table 1:** Summary of the genes/signals regulated by genomic or nongenomic action of TH/THR signal axis

Molecular function	Gene/signal name	Reference
Nongenomic regulation by TH/THR		
Membrane receptor of TH	Integrin αvβ3	[55, 56]
Signal transductor	Src kinase	[59]
	PI3K/Akt	[7, 59, 68]
	p-ERK1/2	[58, 61–66, 60, 67]
	mTOR/p70S6K	[69]
	eNOS	[7, 68]
Transcriptional factor	Estrogen receptor	[62, 67]
	STAT3	[64]
	HIF1-α	[58, 60, 70, 69, 71]
	β-catenin	[77]
Metabolic regulator	GLUT1	[71]
	PFKP	[71]
	MCT 4	[71]
Na-K-ATPase	KCNH2	[72]
Apoptosis regulator	FOXO1	[136, 137, 176]
	BCL2L11	[176]
Genomic regulation by TH/THR		
Transcriptional coregulator of THR	SP1	[48]
	p53	[49]
	Oct-1	[50]
	GHF-1	[51, 52]
	CTCF	[53, 54]
	LCOR	[47]
Autophagy regulator	DAPK2	[16]
	Betatrophin	[26]
Cell cycle regulator	UHRF1	[171]
	STMN1	[172]
	Mir-214	[173]
	*BC200*	[174]
Apoptosis regulator	TRAIL	[175]
Metastatic regulator	BSSP4	[178]
	LCN2	[180]
	mir-130b	[177]
	mir-21	[179]

In addition to positively regulating downstream targets, TH binding to THR can lead to negative regulatory effects. Notably, these negatively regulated target genes are upregulated in the absence but downregulated in the presence of THs [30, 43] although the precise details remain to be established. In a typical case, high levels of THs exert a negative feedback effect on thyrotropin-releasing hormone (TRH) and thyroid stimulating hormone (TSH), and low levels of THs stimulate secretion of TSH from the anterior pituitary. This critical negative feedback loop regulates the hypothalamic-pituitary-thyroid axis [44–46]. TREs of genes negatively regulated by TH are frequently located near proximal promoter regions. However, binding of THRs to these putative regions is generally weak, suggesting that interactions between THRs and other co-factors may contribute significantly to negative regulatory effects of THs. Alterations in chromatin remodeling through histone modification via recruitment of HDACs and HATs may be involved in negative transcriptional regulation by THs. Recently, a novel THR co-repressor, LCOR, was identified as an inhibitor of TRβ-dependent lipogenic gene activity. LCOR serves as a competitor for binding of coactivators SRC-1/3 to TRβ leading to reduced recruitment of SRCs to TREs within the promoter regions of downstream target genes of TR, potentially representing a novel mechanism by which LCOR regulates gene transcription [47].

THRs are additionally reported to interact with transcription factors to negatively regulate expression of several genes, including Sp1 [48], p53 [49], Oct-1 [50], GHF-1 [51, 52] and CTCF [53, 54], although the underlying mechanisms remain largely unknown at present.

#### Nongenomic actions of TH

In addition to transmission of signals through interactions with nuclear THRs, activities of THs in the plasma membrane or cytoplasm are termed nongenomic effects (Fig. 1, Table 1). THs bind to integrin αvβ3 membrane receptor protein independently of nuclear THRs [55–57], which has been further characterized as a membrane-bound THR. Integrin αvβ3 was originally shown to contain the Arg-Gly-Asp (RGD) recognition region that interacts with extracellular matrix ligands [55]. Unexpectedly, TH could bind integrin αvβ3 near its RGD recognition site [58]. Integrin αvβ3 contains two TH-binding domains with no homology to nuclear THRs. The S1 domain mainly recognizes T_3,_ consequently activating the phosphatidylinositol 3-kinase (PI3K)/Akt/protein kinase B (PKB) pathway through Src kinase [59]. Both T_4_ and T_3_ bind to the S2 domain and activate the mitogen-activated protein kinase/extracellular signal-regulated kinase (MAPK/ ERK1/2) pathway. Moreover, S1 and S2 domains mediate the specific effects of TH. For instance, S1 directs Src and PI3K-mediated TRα translocation from the cytoplasm to the nucleus and promotes expression of target genes, such as hypoxia-inducible factor-1α (HIF-1α) while S2 activates MAPK1 and MAPK2, leading to nuclear trafficking of TRβ1 from the cytoplasm and tumor cell proliferation [58, 60].

THs also cause serial phosphorylation and nuclear localization of other critical genes responsible for several cellular functions (Table 1). For instance, TH-activated ERK1/2 has been shown to promote estrogen receptor-α (ERα), signal transducer and activator of transcription-3 (STAT3), and several THR-associated proteins [61–67]. TRα1 interacts with the p85α subunit of PI3K in a T_3_-dependent manner, leading to activation of Akt and endothelial nitric oxide synthase (eNOS) [7, 68]. In addition, liganded TRβ1 associates with p85α in the cytoplasm to activate Akt via phosphorylation. Activated Akt subsequently triggers the nuclear mammalian target of rapamycin (mTOR)-p70S6K cascade and sequential induction of several HIF-1α target genes, including glucose transporter 1 (GLUT1), platelet-type phosphofructokinase (PFKP) and monocarboxylate transporter 4 (MCT 4) [69–71]. TRβ1 is additionally reported to modulate Na^+^/K^+^-ATPase activity by PI3K or ERK1/2. For instance, PI3K signaling slows potassium voltage-gated channel, subfamily H, member 2 (KCNH2) channel deactivation in the plasma membrane of pituicytes [72–74]. Moreover, THRB-PV, a THRB mutant that shows loss of T_3_ binding ability but interacts more significantly with the PI3K regulatory subunit, p85, triggers a greater increase in PI3K kinase activity and activation of the PI3K-AKT- mTOR-p70S6K pathway in cytoplasmic and nuclear compartments, with predisposition to tumor development in several cancer types, including thyroid and mammary tumors [75, 76]. The THRB-PV mutant additionally associates with β-catenin to regulate cell proliferation in thyroid tumors of THRB ^PV^/ ^PV^ mice [77]. This interaction favors the unliganded state of TRβ, and T_3_-independent interactions between β-catenin and TRβ promote activation of β-catenin-related downstream targets.

### The TH/THR axis in regulation of hepatic autophagy

#### The autophagic process

Autophagy was originally characterized as a catabolic process targeting cellular constituents, including unfolded proteins, damaged organelles and intracellular pathogens, to lysosomes for degradation [19, 78]. Autophagy is categorized into three main types: macroautophagy, chaperone-mediated autophagy and microautophagy [79]. Macroautophagy, hereafter known as autophagy, is generally considered the major route for directing cytoplasmic components into lysosomes for degradation. The autophagic process involves membrane biogenesis and formation of a double-membrane phagophore (termed autophagosome), which sequesters partial cytoplasmic components or entire organelles and subsequently fuses with lysosomes for degradation. Amino acids and other metabolic compounds generated by this process are consequently released for energy production or recycling. Chaperone-mediated autophagy involves sequestration of proteins or polypeptides harboring the KFERQ-like motif by chaperone proteins. This process promotes translocation of target proteins into lysosomes for degradation through interactions with lysosome-associated membrane protein type 2A (LAMP2A). Microautophagy is implicated in invagination of cellular constituents within endosomes or lysosomes but small fractions of cytoplasmic constituents in the close vicinity of lysosomes are sequestered.

Under basal conditions, autophagy is implicated in the degradation of long-lived proteins while another catabolic system, the ubiquitin-proteasome process, is responsible for the turnover of short-lived proteins [80, 81]. However, under specific conditions, such as nutrient deprivation, the autophagy pathway leads to selective degradation of cytosolic materials (termed selective autophagy). Selective autophagy directs degraded products into highly spatiotemporally controlled metabolic pathways. When specific autophagic cargo, such as misfolded proteins or damaged cellular organelles, appear within the cytoplasm, they are tagged with molecular markers, such as ubiquitin [82, 83], resulting in assembly of autophagic adapter proteins, such as SQSTM1, that bind to both molecular marker-harboring cargo and LC3-II. A number of core autophagy proteins, such as the ULK-FIP200 complex, also recognize these tagged targets [84, 85], initiating autophagosome formation. Selective autophagy is predominantly regulated by cargo labeling as well as recruitment of adaptor proteins to cargo.

Significant links between the regulation of selective autophagy and liver complications associated with NAFLD and HCC have been reported, supporting the manipulation of this process as a potential therapeutic strategy for liver-related diseases.

#### Autophagy in liver-related diseases

In addition to the fundamental function of starvation-induced autophagy, basal and selective autophagy contribute to maintaining the quality and quantity of cellular organelles and cytosolic proteins efficiently in the liver. Consequently, dysregulation or malfunction of the autophagic process is associated with the pathogenesis of multiple disorders and liver-related diseases, such as age-related hepatic disorders, NAFLD and HCC [86].

##### Aging in liver

Aging is positively associated with severity and poor prognosis of several liver-related diseases, including alcoholic liver disease, NAFLD and HCC [87]. Furthermore, the age-dependent frequent decrease in autophagic activity underlies the pathogenesis of hepatic diseases. The initial finding of age-dependent decrease in hepatic autophagy was based on a marked increase in oxidative damage-triggered protein carbonyl derivatives in liver of 27-month-old rats, compared to 2-month-old rats [88]. Further studies indicated that the efficiency of autophagic degradation and capacity of autophagic proteolysis of exogenous amino acids of primary hepatocytes from older rats is dramatically decreased relative to that in young rats [89, 90]. Moreover, decreased expression of LC3-II and number of autophagosomes in mice were age-dependent [91]. These results suggest that the age-dependent decrease in efficiency of autophagy leads to substantially diminished clearance of inactive organelles, including mitochondria, generating increased oxidative stress and consequent accumulation of oxidized protein aggregates.

##### NAFLD

Fatty liver is attributed to continuous intake of excess dietary fat without consumption of excessive alcohol [92]. Nonalcoholic fatty liver disease (NAFLD) incorporates a spectrum of liver-related diseases ranging from steatosis to steatohepatitis, fibrosis and cirrhosis. Non-alcoholic steatohepatitis (NASH) presents as a hepatic disease histologically similar to alcoholic hepatitis but occurs without consumption of excessive alcohol, representing a stage within NAFLD [93, 94]. Recent metabolic studies on animals and humans demonstrated that NAFLD represents one feature of metabolic syndrome closely associated with several metabolic diseases, such as diabetes and insulin resistance. Moreover, diabetes or insulin resistance conditions accelerate the entire pathological spectrum of NAFLD [94]. Chronic hepatic steatosis can trigger inflammatory responses [95]. In some cases, NAFLD progresses to NASH, which frequently advances into fibrosis and cirrhosis, and 4–27% NASH cases develop HCC [96].

In response to accelerated lipid availability or nutrient starvation, hepatic autophagy degrades lipid droplets to produce free fatty acids (FFA) for ATP generation. This autophagy-induced degradation of hepatic lipid droplets is termed lipophagy [97]. Since lipophagy involves the selective degradation of hepatic lipid droplets, autophagy in liver could serve as a preventive mechanism against NAFLD. In contrast, several studies indicate that lipotoxic effects, including oxidative stress or insulin resistance, elicited by excess triglycerides and free fatty acids in NAFLD, inhibit activation of autophagy [19, 98, 99]. Hepatic autophagy regulates lipid metabolism through elimination of triglyceride accumulation in liver and prevents the development of steatosis [97]. Enhancement of autophagic activity using pharmaceutical agents, such as rapamycin or carbamazepine, has been shown to retard liver steatosis [99–102]. Moreover, pharmacological inhibition of autophagy via 3-methyladenine or knockdown of the essential autophagy gene, *atg5,* in hepatocytes challenged with a lipid load induced a dramatic increase in the cellular triglyceride level. Excessive triglyceride and cholesterol ester accumulation in hepatic lipid droplets was observed owing to decreased lipolysis and fatty acid 훽-oxidation in cells with low autophagy activity.

Compared to hepatocytes, autophagy in stellate cells exerts opposite effects on NAFLD progression. In NAFLD, quiescent hepatic stellate cells are activated and transdifferentiate into myofibroblasts, which express a large number of inflammatory cytokines and collagen, thereby promoting hepatic fibrosis [103]. In stellate cells from livers of autophagy-deficient mice, CCl_4_-induced hepatic fibrosis was dramatically inhibited [104].

Abnormal structural and functional alterations of hepatic mitochondria in NAFLD are frequently observed [105]. Mitochondria are the powerhouse of cells and decreased mitochondrial function concomitant with alterations in structural and molecular pathways may elicit a metabolic imbalance, contributing to NAFLD progression. Mitochondrial biogenesis and mitophagy, a highly selective form of autophagy that functions in removal of damaged mitochondria, are the major pathways that regulate mitochondrial mass [86, 105]. The balance of mitochondrial biogenesis and mitophagy is a precisely regulated process that influences cellular homeostasis. Activation of hepatic mitophagy is reported to eliminate the lipid content and oxidative stress, and dysregulation of mitophagy implicated in the progression of NAFLD [19, 86, 97].

Under oxidative stress conditions, SQSTM1 is phosphorylated and subsequently binds to KEAP1 with high affinity. KEAP1 is an adaptor of the ubiquitin ligase complex for nuclear factor-erythroid 2-related factor-2 (NRF2). Downstream target proteins of NRF2, such as NAD(P)H, dehydrogenase quinone 1 (NQO1) and glutathione *S*-transferase (GST), ameliorate ROS production by damaged mitochondria [106, 107]. Selective autophagic degradation of the SQSTM1-KEAP1 complex inhibits KEAP1-driven ubiquitylation and degradation of NRF2. In NAFLD, the turnover of hepatic cytoplasm fractions is substantially impaired due to dysfunctional autophagy/mitophagy, leading to accumulation of damaged mitochondria and elevated oxidative stress, which activates the SQSTM1–KEAP1–NRF2 pathway to protect hepatocytes against oxidative stress. However, under conditions where ROS levels exceed the antioxidant capacity of NRF2-related signals, various harmful effects, including lipid peroxidation, protein oxidation, and DNA damage, trigger liver injury [106, 107].

##### HCC

As autophagy plays important roles in maintenance of the quality of organelles and supply of energy to cancer cells, autophagy-related pathways are considered important for cancer cell survival [108]. Previous studies indicate that loss of autophagy inhibits KRAS–triggered tumorigenesis of non small-cell lung cancer [109, 110]. Indeed, several clinical trials using a combination of existing anticancer drugs and autophagy inhibitors, such as chloroquine and hydroxychloroquine, are currently underway for several cancer types [111, 112]. However, the specific functions of autophagy in different tumors are complex and context-dependent. Pancreas-specific activated KRAS in mice leads to the development of pancreatic ductal adenocarcinoma (PDAC), which is suppressed by inhibition of autophagy [113]. In contrast, in mice lacking *Tp53*, loss of autophagy facilitates tumor progression [113].

In the liver, autophagy appears to function as a tumor suppressor. For instance, mosaic depletion of *Atg5*, liver-specific *Atg7*, or *Beclin-1* in mice causes accumulation of degenerated protein aggregates, lipid droplets and damaged cellular organelles, including mitochondria and peroxisomes, as well as persistent activation of NRF2 owing to sequestration of KEAP1 by SQSTM1-positive cytoplasmic aggregates, leading to spontaneous hepatic carcinogenesis [114–116]. Simultaneous loss of nrf-2 or sqstm1 in mice with *Atg5* or *Atg7*-deficient liver suppresses tumor development [117]. Additionally, the cargo receptor degraded by autophagy, SQSTM1, accumulates in the hepatic tumor region [118], implying that the SQSTM1–KEAP1–NRF2 axis contributes to tumor growth. Further studies have revealed that heterozygous deletion of a major regulator of autophagy, *Beclin1*, increases the frequency of development of spontaneous tumors and HBV–induced hepatic premalignant lesions in mice [116, 119]. Additionally, ATG5 and BECLIN-1 levels are downregulated in hepatic tumor, compared to adjacent non-tumor regions [120]. HCC patients with low BECLIN-1 accompanied by high Bcl-xL (a crucial anti-apoptotic protein) expression display poorer disease-free and overall survival rates [120], indicating that normal autophagic flux is important for HCC prevention in this apoptosis compromised background.

In view of the protective function of autophagy against hepatocarcinogenesis, researchers have focused on the mechanisms underlying autophagy-dependent tumor cell death identified in several cancer types [121–123]. The PI3K/Akt/mTOR axis is a known crucial signaling pathway for cell growth, survival and metabolism in tumor cells [124]. The mTOR pathway is activated in HCCs and manipulation of mTOR inhibitors shown to effectively exert anti-tumor effects in HCC [125, 126]. Rapamycin and its derivatives are mTOR inhibitors reported to serve as autophagy inducers with anti-tumor activity in a phase II study on 25 advanced HCC patients [127]. Liver transplantation is an important therapeutic option for the selected patients with unresectable HCC. In another study, rapamycin-directed immunosuppression was associated with improved survival after liver transplantation in HCC patients, but showed a trend toward lower survival in non-HCC patients, further showing the clinical evidence of its anti-cancer impact [128]. However, the utility of rapamycin and its derivatives in HCC therapy is controversial due to insufficient and conflicting clinical results. For example, everolimus (RAD001) exerted an anti-tumor effect in xenografts of human HCC models [129] whereas a recent clinical phase III trial disclosed no benefits on advanced HCC prognosis [130]. Co-targeting of mTOR via everolimus along with a PI3K/mTOR dual inhibitor, BEZ235, displayed greater efficacy through activating autophagy, specifically mitophagy, in tumors and led to decreased tumor sizes in a mouse model of HCC [131]. Interestingly, recent findings suggest that combination of mTOR inhibitors with SBI-0206965, a highly selective ULK1 inhibitor acting as a specific blocker of autophagy, has a promising effect on HCC [132]. Further investigations are required to validate the clinical utility of rapamycin. Sorafenib, a multi-kinase inhibitor used as first-line systemic therapy for advanced HCC, promotes autophagy-dependent cell death through Mcl-1 signaling [127]. A combination of sorafenib and autophagy inhibitors was also shown to induce an enhanced therapeutic effect. Sorafenib-induced autophagy-dependent cell death is reported to cause drug resistance in HCC [133]. Further research is therefore warranted to determine the utility of autophagy inducers in improving the current limits of HCC therapy and treatment outcomes. The involvement of autophagy as a function of tumor type, pathological stage and genetic context remains to be established.

#### TH/THR regulation of hepatic autophagy

The effects of TH on hepatic lysosomal activity and proteolysis were first described in 1978 [134], although the underlying mechanisms were yet to be elucidated. Recently, T_3_ was shown to enhance hepatic lysosomal activity accompanied by formation of autophagosomes in hepatic cells or livers of mice [25, 26]. The effects of T_3_ on autophagy were THR-dependent and binding of NCoR-HDAC3, the corepressor of THR, abolished T_3_-induced hepatic autophagy [25].

**Fig. 2:**
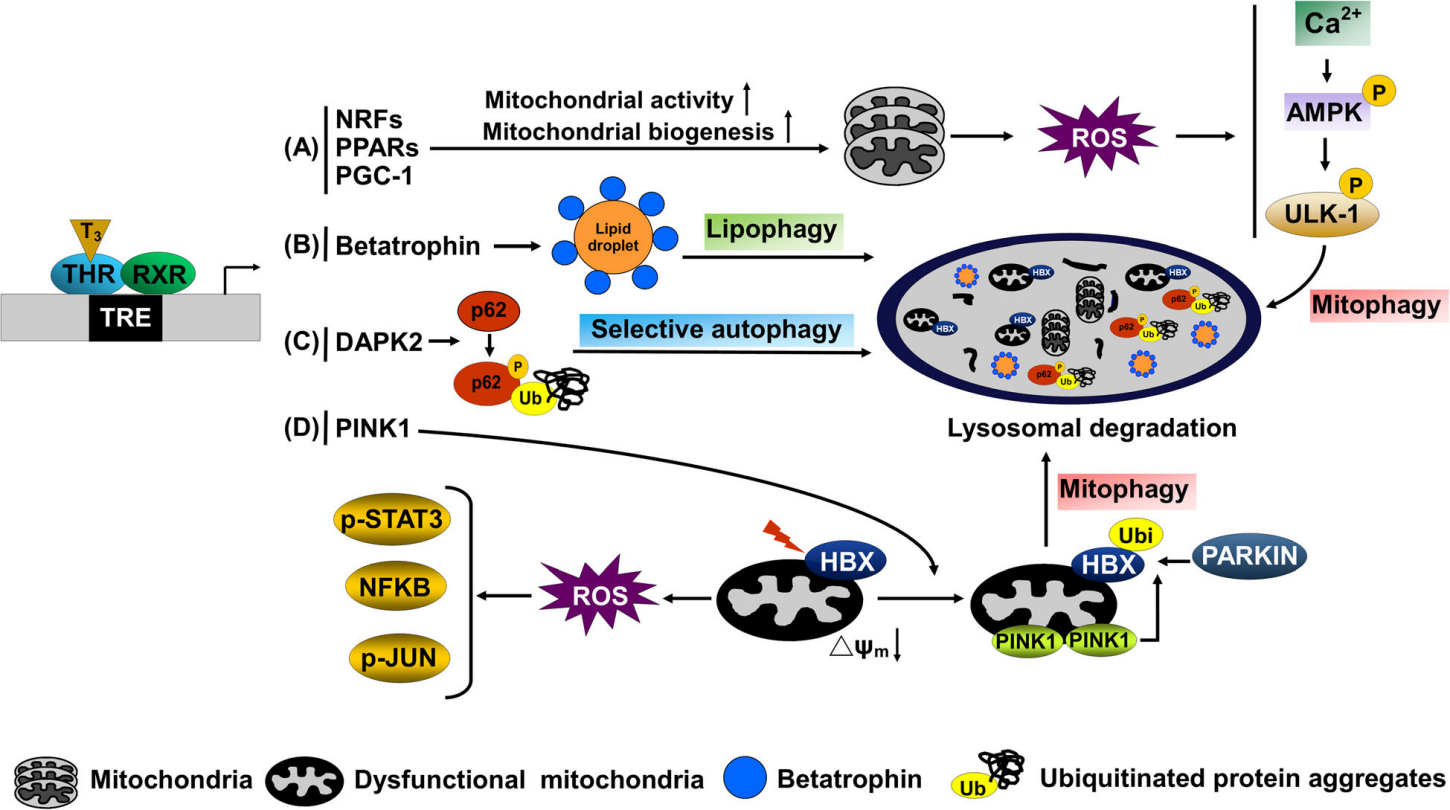
Model to Thyroid hormone stimulated hepatic autophagy. **a** T_3_**/**THR promotes mitochondrial activity and biogenesis through inducing transcriptional regulators, such as PPARs, PPAR coactivators (PGC-1) and nuclear respiratory factors (NRF), causing reactive oxygen species (ROS) generation and subsequently release of intracellular calcium, and ultimately, stimulating AMPK-ULK-1 dependent mitophagy. **b** T3/THR upregulates at transcriptional level betatrophin to promote lipophagy. **c** T3/THR induces DAPK2-mediated of SQSTM1 (p62) phosphorylation to enhance selective autophagy **d** HBV X protein (HBx) targets to mitochondria and consequently causes ROS generation, implicated in activation of STAT-3, JNK and NF-κB. T_3_**/**THR activates PINK1/PARKIN-dependent mitophagy to ameliorate HBX-induced mitochondrial depolarization

The T_3_/THR axis is known to promote fatty acid 훽-β-oxidation in liver via activation of autophagy (Fig. 2A). Additionally, THs upregulate several critical genes involved in the autophagic process, including ULK1, PINK1, Beclin-1, DAPK2, betatrophin and LC3 (Fig. 2B-D) [16, 17, 25–27, 135]. These autophagy-related genes could be regulated directly by T_3_/THR at the transcriptional level or indirectly through FOXO1 activation by dephosphorylation and deacetylation via TH-activated SIRT1 [136, 137]. SIRT1 is a NAD^+^-dependent deacetylase activated by increases in cellular NAD^+^ levels that serves as an energy sensor of cells to control transcriptional activity by T_3_ and FOXO1. Furthermore, SIRT1 mediates T_3_-induced autophagy through stimulation of expression as well as deacetylation of autophagy-related genes [136]. The master transcription factor, transcription factor EB (TFEB), regulates autophagy and lysosome-related genes may additionally be modulated by TH [44]. Other than transcriptional regulation, TH/THR complexes also regulate autophagy through post-transcriptional mechanisms. For instance, TH/THR is reported to activate the autophagy process through AMPK signaling. T_3_ induces mitochondrial activity and biogenesis through inducing transcriptional regulators, such as PPARs, PPARγ coactivator-1 (PGC-1) and nuclear respiratory factors [138], which causes the generation of reactive oxygen species (ROS) and subsequently release of intracellular calcium, and ultimately, CAMKK2 activation. Activated CAMKK2 phosphorylates AMPK, in turn, inhibiting mTOR signaling and stimulating autophagy via ULK1 phosphorylation [27]. SQSTM1 is a key adapter protein of autophagy, and accumulating evidence has demonstrated that phosphorylation of this protein facilitates clearance of ubiquitinated protein aggregates through the autophagic process [139, 140]. We previously showed that T_3_/THR interactions induce transcription of DAPK2, which, in turn, phosphorylates SQSTM1 to promote clearance of protein aggregates through autophagy. Our results collectively indicate that the TH/THR signaling axis coordinates both transcriptional and post-translational regulation of hepatic autophagy [16].

##### TH regulation of selective autophagy

Lipophagy is implicated in the digestion of neutral lipid droplets to release free fatty acids for mitochondrial lipid oxidation [97] and considered a major lipolytic pathway in hepatic cells [141]. Recently, we and other groups showed that T_3_ induces lipophagy in both human and mouse hepatic cells that is essential for T_3_-mediated lipid catabolism [25, 26]. Although the specific receptors for recognition of lipid droplets by autophagosomes have not been identified, our results indicate that T_3_ upregulates hepatic betatrophin transcription, which localizes to lipid droplets and possibly targets lipids for autophagic degradation [26].

TH is considered an inducer of mitochondrial activity and oxidative stress in the liver [142]. However, TH also induces mitophagy to prevent accumulation of damaged mitochondria and prevents hepatic injury by excessive ROS production [27]. TH is reported to stimulate the AMPK-ULK1 axis to initiate mitophagy. This process is dependent on translocation of ULK1 to mitochondria and subsequent recruitment of autophagic proteins, such as SQSTM1 and LC3-II, to damaged mitochondria for eventual degradation in autolysosomes. PINK1/PARKIN-mediated mitophagy is another pathway that contributes to protecting mitochondria against cellular ROS [143, 144]. PINK1 accumulates at the outer mitochondrial membrane upon membrane depolarization and subsequently recruits PARKIN to ubiquitinate damaged mitochondria, facilitating autophagic degradation. Experiments by our group showed that T_3_ activates PINK1/PARKIN-dependent mitophagy to ameliorate HBX-induced mitochondrial depolarization [17]. Selective removal of damaged mitochondria by TH is crucial for preventing oxidative damage in the liver.

Recently, we demonstrated that transcriptional regulation of DAPK2 by TH promotes phosphorylation of SQSTM1 to facilitate clearance of diethylnitrosamine (DEN)-induced protein aggregates through autophagy, which may protect hepatocytes from DEN-induced hepatocarcinogenesis [16].

### Potential application of TH and analogs in NAFLD and HCC

Thyroid hormones affect energy metabolism, glucose homeostasis and lipid utilization. Hypothyroidism is positively associated with high risk of NAFLD and HCC incidence, independent of other risk factors [8, 9]. THs may thus be useful in a therapeutic capacity in hyperlipidemia and NAFLD. However, excessive production or administration of exogenous THs triggers several side- effects, such as muscle wasting, increased heart rate with possible atrial arrhythmia, and heart failure [145]. Over the past few decades, biologists have focused on the possibility that TH derivatives have the beneficial actions of the thyroid hormone but without associated deleterious effects [145–149]. Recently, several analogs of TH specific for THRB have been generated, which have therapeutic activity in liver-related diseases with limited side-effects in organs, such as heart or bone, with abundant THRA expression.

GC-1 was the first synthetic THRB agonist that could be used as a scaffold compound for developing other TH derivatives, which are easily modified and synthesized more efficiently than native TH [168]. GC-1 binds all major isoforms of THRB with similar affinity to T_3._ The binding affinity of GC-1 to THRB is 10-fold higher than that to THRA. GC-1 accumulates predominantly in the liver but its uptake is low in other organs, including skeletal muscle and heart. Due to the specific binding of GC-1 to THRB in liver, it may exhibit gene-specific actions relative to the native form of the thyroid hormone [145].

In the CMD diet-triggered NAFLD rat model, administration of either T_3_ or GC-1 could prevent steatohepatitis. Notably, GC-1 treatment not only caused a more significant reduction in hepatic TG levels but also did not elicit significant side-effects, such as increased heart rate and muscle wasting [150, 151]. These findings support the potential therapeutic application of THs on NAFLD prevention. Furthermore, T_3_ and GC-1 exert therapeutic effects on HCC [16, 29, 152, 153]. Upon treatment of rats with DEN combined with a choline-deficient (CD) diet for weeks, development of preneoplastic lesions was observed. Administration of T_3_ or GC-1 dramatically reduced the preneoplastic lesions caused by DEN.

Recently, a liver-selective prodrug, MB07811, was developed. Following hepatic enzymatic cleavage, the active form, MB07344, is generated that has been characterized as a liver-selective THRB agonist [154]. In HFD-exposed or diabetic fatty animals, two weeks of MB07811 treatment significantly reduced both hepatic and plasma triglyceride levels with no other side-effects of TH [154]. Clearance of hepatic lipid droplets by MB07344 may be attributable to acceleration of mitochondrial activity and fatty acid catabolism [12].

KB2115 has been identified as another THRB-selective agonist preferentially taken up in the liver. In both animal and clinical studies, administration of KB2115 significantly lowered serum total and LDL cholesterol and prevented the development of hepatic steatosis [155–157]. Furthermore, treatment with KB2115 as well as GC-1 in rats induced hepatomitogenic activity with no evidence of hepatic toxicity [158], supporting its potential for regenerative therapy, including liver transplantation and other surgical modalities.

Despite encouraging results from human clinical studies showing that GC-1, MB07811 and KB2115 exert therapeutic effects via lowering the levels of serum LDL cholesterol and triglycerides, these compounds have not reached human clinical trials or been developed into therapeutic agents. Phase II trials on GC-1 and MB07344 are yet to be performed. Clinical studies on KB2115 were discontinued due to cartilage damage and hepatic toxicity observed following long-term dosing in dogs [29, 159].

More recently, two liver-directed THRB selective agonists, MGL-3196 and VK2809, have been developed [160, 161]. Results from phase II trials showed preventive effects on NAFLD accompanied by a decrease in serum levels of LDL cholesterol and triglycerides as well as hepatic lipids with none of the side-effects of the thyroid hormone axis. Thus, therapeutic application of THs in liver-related diseases in the clinic is feasible.

Recent studies by our group revealed a mechanistic link between TH and HCC prevention [16, 17]. DEN-treated liver cells have been shown to cause ROS accumulation accompanied by increased DNA damage and hepatic injury [162]. Increased oxidative stress may occur due to the accumulation of SQSTM1-associated protein aggregates and damaged organelles. SQSTM1 is the major component of inclusion bodies in hepatocytes (termed Mallory bodies), which have been identified in the livers of patients diagnosed with alcoholic hepatitis and NAFLD [163, 164]. T_3-_treated mice exhibit higher DAPK2 expression, and consequently, T_3_-driven autophagy alleviates DEN triggered hepatic injury and hepatocellular carcinogenesis [16].

Chronic infection of hepatitis B virus in liver is one of the major risk factors for HCC development, and the HBV X protein (HBx) exerts powerful disruptive effects on mitochondrial dysfunction and ROS production, leading to progression of HCC [165]. Our group further showed that mitophagy triggered by the TH-PINK1-Parkin axis is a putative pathway implicated in protection of HBx-induced hepatocellular carcinogenesis. Additionally, TH-triggered autophagy was shown to reduce hepatic lipid droplets and mitochondrial fatty acid oxidation [25, 27]. These results collectively support the involvement of TH-triggered autophagy in regulating mitochondrial metabolism in the development of NAFLD and HCC and provide insights into the physiological significance of THs in prevention of liver-related diseases [17]. However, the role of autophagy in the preventive and therapeutic potential of TH analogs (GC-1, MB07344, KB2115, MGL-3196 and VK2809) have not yet been determined.

## Conclusions

Over the past decade, molecular mechanisms and physiological effects of THs in liver have gradually been elucidated. Disruption of TH signals is known to cause multiple organ dysfunction that is closely associated with several diseases [5, 6]. Liver is one of the major target tissues of TH, and people with low thyroid function are closely associated with multiple liver-related diseases. The cross-sectional and systemic view studies indicated that subclinical hypothyroidism, and even in the upper normal limit of TSH levels were significantly associated with the risk of NAFLD and advanced fibrosis [166–169]. Interestingly, high level of TSH itself may be an important risk factor points to the pathogenesis of NAFLD, independent of thyroid hormones [168, 169], and the supplementation of levothyroxine shows clear benefits on NAFLD in subclinical- and mild subclinical- hypothyroidism patients with dyslipidemia [170]. Moreover, both in *vitro* and in *vivo* experiments demonstrated THs and THs analogs exhibit the potential therapeutic and preventive application in NAFLD and HCC [7, 16, 17, 142, 145–149], highlighting the urgent need to understand the complex mechanisms underlying the effects of thyroid hormone. The TH/THR axis is a strong inducer of hepatic autophagy, which promotes lipid droplet degradation as well as mitochondrial biogenesis and turnover. This process has been implicated in the removal of damaged mitochondria and ROS that cause hepatic injury [7, 16, 17, 26, 27, 28]. In the present review, we have discussed the growing complexity of TH-regulated autophagy, highlighted advantages associated with the TH/autophagy axis-based therapeutic strategy for liver-related diseases, and discussed recent findings that may be exploited for improving the therapeutic outcomes of NAFLD and HCC [11, 12, 148, 150, 151, 154].

However, several challenges in TH-based therapies for hepatic diseases, HCC in particular, remain to be overcome, since the actions of individual TH derivatives within the same tissue may be different. Alterations in TH deiodinases, transporters, co-activators or co-repressors may influence the cellular level and molecular actions of THs, in turn, causing metabolic changes [44]. Moreover, the processes of cancer progression are complex. Individual THR isoforms exert different effects in a cancer type- and stage-specific manner. The TH/THR signals and interacting partners may facilitate the switch from tumor suppression in the premalignant stages to promotion in the later stages of HCC [7]. For instance, administration of TH not only reduces the size of preneoplastic lesions in the livers of rats suffering with HCC, but suppresses the aberrant cellular growth via control the expression of cell cycle regulators, such as CDK2, Cyclin E, UHRF1, STMN1 mir-214 and *BC200* lncRNA [7, 171–174]. Our recent studies further support the preventive effect of TH on hepatocarcinogenesis via activating autophagy [16, 17], whereas TH/THR is reported to promote metastasis and chemoresistance through control the expressions of BSSP4, TRAIL, BCL2L11, LCN2, mir-21, and mir-130b [7, 173, 175–180]. This characteristic of THs supports the double-edged sword effect of autophagy in cancer progression. Autophagy mitigates stress-caused damage by removing damaged cellular organelles and protein aggregates and impaired autophagy causes accumulation of excess oxidative stress and DNA damage, leading to initiating hepatocarcinogenesis. Mosaic depletion of *Atg5*, liver-specific *Atg7* or *Beclin-1* in mice causes accumulation of degenerated protein aggregates, lipid droplets and damaged cellular organelles, leading to spontaneous hepatic carcinogenesis [114, 116]. By contrast, after the initiation of tumorigenesis, autophagy can also facilitate tumor cell survival under metabolic stress, becoming dormant and regenerating with anti-stress capacity that promotes tumor progression [181]. For instance, malfunction of autophagy inhibits KRAS–triggered tumorigenesis of non small-cell lung cancer and DEN-induced HCC. [109, 110, 182]. Moreover, inhibition of autophagy increases the sensitivity of chemotherapy and triggers cellular apoptosis and necrosis of HCC by activating several tumor suppressor genes, including p53, PTEN CDKN1, CDKN2 and Rb1 [182–184]. Therefore, further animal and clinical studies are warranted to establish the specific functions of THs-induced autophagy in the different processes that lead to HCC development.

## Abbreviations

CD: Choline-deficient
DEN: Diethylnitrosamine
eNOS: Nitric oxide synthase
ERK: Extracellular signal-regulated kinase
ERα: Estrogen receptor-α
GLUT1: Glucose transporter 1
GST: Glutathione *S*-transferase
DIO: Iodothyronine deiodinase
HAT: Histone acetyl transferase
HCC: Hepatocellular carcinoma
HDAC: Histone deacetylase
HFD: High-fat diet
HIF-1α: Hypoxia-inducible factor-1α
KCNH2: Potassium voltage-gated channel, subfamily H, member 2
LAMP2A: Lysosome-associated membrane protein type 2A
MAPK: Mitogen-activated protein kinase
MCT 4: Monocarboxylate transporter 4
mTOR: Mammalian target of rapamycin
NAFLD: Non-alcoholic fatty liver disease
NCoR1: Nuclear receptor corepressor 1
NQO1: NAD(P)H, dehydrogenase quinone 1
NRF2: Nuclear factor-erythroid 2-related factor-2
PCAF: p300/CBP-associated factor
PDAC: Pancreatic ductal adenocarcinoma
PFKP: Platelet-type phosphofructokinase
PGC-1: PPARγ coactivator-1
ROS: Reactive oxygen species
RGD: Arg-Gly-Asp
RXR: Retinoid X receptor
SMRT: Silencing mediator for retinoid or thyroid-hormone receptors
SRC: Steroid hormone receptor coactivator
STAT3: Signal transducer and activator of transcription-3
TFEB: Transcription factor EB
TH: Thyroid hormone
THR: Thyroid hormone receptor
TRAP: TR-associated protein
TRE: Thyroid hormone response elements
TRH: Thyrotropin-releasing hormone
TSH: Thyroid stimulating hormone
VDR: Vitamin D receptors

## References

[1] Yen PM (2001). Physiological and molecular basis of thyroid hormone action. Physiol Rev.

[2] Sinha RA, Singh BK, Yen PM. Direct effects of thyroid hormones on hepatic lipid metabolism. Nat Rev Endocrinol. 2018;14(5):259–69. 10.1038/nrendo.2018.10 PMC6013028 29472712

[3] Feely J, Isles TE (1979). Screening for thyroid dysfunction in diabetics. Br Med J.

[4] Gray RS, Irvine WJ, Clarke BF (1979). Screening for thyroid dysfunction in diabetics. Br Med J.

[5] Tatar E, Kircelli F, Asci G, Carrero JJ, Gungor O, Demirci MS (2011). Associations of triiodothyronine levels with carotid atherosclerosis and arterial stiffness in hemodialysis patients. Clin J Am Soc Nephrol..

[6] Tatar E, Sezis Demirci M, Kircelli F, Gungor O, Yaprak M, Asci G (2012). The association between thyroid hormones and arterial stiffness in peritoneal dialysis patients. Int Urol Nephrol.

[7] Chi HC, Chen CY, Tsai MM, Tsai CY, Lin KH (2013). Molecular functions of thyroid hormones and their clinical significance in liver-related diseases. Biomed Res Int.

[8] Hassan MM, Kaseb A, Li D, Patt YZ, Vauthey JN, Thomas MB (2009). Association between hypothyroidism and hepatocellular carcinoma: a case-control study in the United States. Hepatology.

[9] Lee J, Ha J, Jo K, Lim DJ, Lee JM, Chang SA (2018). Male-specific association between subclinical hypothyroidism and the risk of non-alcoholic fatty liver disease estimated by hepatic steatosis index: Korea National Health and Nutrition Examination Survey 2013 to 2015. Sci Rep.

[10] Liangpunsakul S, Chalasani N (2003). Is hypothyroidism a risk factor for non-alcoholic steatohepatitis?. J Clin Gastroenterol.

[11] Perra A, Simbula G, Simbula M, Pibiri M, Kowalik MA, Sulas P (2008). Thyroid hormone (T3) and TRbeta agonist GC-1 inhibit/reverse nonalcoholic fatty liver in rats. FASEB J.

[12] Cable EE, Finn PD, Stebbins JW, Hou J, Ito BR, van Poelje PD (2009). Reduction of hepatic steatosis in rats and mice after treatment with a liver-targeted thyroid hormone receptor agonist. Hepatology.

[13] Akino K, Akita S, Mizuguchi T, Takumi I, Yu R, Wang XY (2005). A novel molecular marker of pituitary tumor transforming gene involves in a rat liver regeneration. J Surg Res.

[14] Mollica MP, Lionetti L, Moreno M, Lombardi A, De Lange P, Antonelli A (2009). 3,5-diiodo-l-thyronine, by modulating mitochondrial functions, reverses hepatic fat accumulation in rats fed a high-fat diet. J Hepatol.

[15] Naehrlich L, Dorr HG, Bagheri-Behrouzi A, Rauh M. Iodine deficiency and subclinical hypothyroidism are common in cystic fibrosis patients. J Trace Elem Med Biol. 2012. 10.1016/j.jtemb.2012.08.002 23107148

[16] Chi HC, Chen SL, Tsai CY, Chuang WY, Huang YH, Tsai MM (2016). Thyroid hormone suppresses hepatocarcinogenesis via DAPK2 and SQSTM1-dependent selective autophagy. Autophagy.

[17] Chi HC, Chen SL, Lin SL, Tsai CY, Chuang WY, Lin YH (2017). Thyroid hormone protects hepatocytes from HBx-induced carcinogenesis by enhancing mitochondrial turnover. Oncogene.

[18] Klionsky DJ, Abdelmohsen K, Abe A, Abedin MJ, Abeliovich H, Acevedo Arozena A (2016). Guidelines for the use and interpretation of assays for monitoring autophagy (3rd edition). Autophagy.

[19] Yang L, Li P, Fu S, Calay ES, Hotamisligil GS (2010). Defective hepatic autophagy in obesity promotes ER stress and causes insulin resistance. Cell Metab.

[20] Stolz A, Ernst A, Dikic I (2014). Cargo recognition and trafficking in selective autophagy. Nat Cell Biol.

[21] Feng GS (2012). Conflicting roles of molecules in hepatocarcinogenesis: paradigm or paradox. Cancer Cell.

[22] Ding WX (2010). Role of autophagy in liver physiology and pathophysiology. World J Biol Chem.

[23] Komatsu M (2012). Liver autophagy: physiology and pathology. J Biochem.

[24] Cui J, Gong Z, Shen HM (2013). The role of autophagy in liver cancer: molecular mechanisms and potential therapeutic targets. Biochim Biophys Acta.

[25] Sinha RA, You SH, Zhou J, Siddique MM, Bay BH, Zhu X (2012). Thyroid hormone stimulates hepatic lipid catabolism via activation of autophagy. J Clin Invest.

[26] Tseng YH, Ke PY, Liao CJ, Wu SM, Chi HC, Tsai CY (2014). Chromosome 19 open reading frame 80 is upregulated by thyroid hormone and modulates autophagy and lipid metabolism. Autophagy.

[27] Sinha RA, Singh BK, Zhou J, Wu Y, Farah BL, Ohba K (2015). Thyroid hormone induction of mitochondrial activity is coupled to mitophagy via ROS-AMPK-ULK1 signaling. Autophagy.

[28] Senese R, Cioffi F, de Lange P, Goglia F, Lanni A (2014). Thyroid: biological actions of 'nonclassical' thyroid hormones. J Endocrinol.

[29] Kowalik MA, Columbano A, Perra A (2018). Thyroid Hormones, Thyromimetics and Their Metabolites in the Treatment of Liver Disease. Front Endocrinol.

[30] Brent GA (2012). Mechanisms of thyroid hormone action. J Clin Invest.

[31] Weinberger C, Thompson CC, Ong ES, Lebo R, Gruol DJ, Evans RM (1986). The c-erb-A gene encodes a thyroid hormone receptor. Nature.

[32] Sap J, Munoz A, Damm K, Goldberg Y, Ghysdael J, Leutz A (1986). The c-erb-A protein is a high-affinity receptor for thyroid hormone. Nature.

[33] Cheng SY, Leonard JL, Davis PJ (2010). Molecular aspects of thyroid hormone actions. Endocr Rev.

[34] Williams GR (2000). Cloning and characterization of two novel thyroid hormone receptor beta isoforms. Mol Cell Biol.

[35] Mitsuhashi T, Tennyson GE, Nikodem VM (1988). Alternative splicing generates messages encoding rat c-erbA proteins that do not bind thyroid hormone. Proc Natl Acad Sci U S A.

[36] Sakurai A, Nakai A, DeGroot LJ (1989). Expression of three forms of thyroid hormone receptor in human tissues. Mol Endocrinol.

[37] Ayers S, Switnicki MP, Angajala A, Lammel J, Arumanayagam AS, Webb P (2014). Genome-wide binding patterns of thyroid hormone receptor beta. PLoS One.

[38] Grontved L, Waterfall JJ, Kim DW, Baek S, Sung MH, Zhao L (2015). Transcriptional activation by the thyroid hormone receptor through ligand-dependent receptor recruitment and chromatin remodelling. Nat Commun.

[39] Ramadoss P, Abraham BJ, Tsai L, Zhou Y, Costa-e-Sousa RH, Ye F (2014). Novel mechanism of positive versus negative regulation by thyroid hormone receptor beta1 (TRbeta1) identified by genome-wide profiling of binding sites in mouse liver. J Biol Chem.

[40] Cheng SY (2000). Multiple mechanisms for regulation of the transcriptional activity of thyroid hormone receptors. Rev Endocr Metab Disord.

[41] McKenna NJ, O'Malley BW (2002). Combinatorial control of gene expression by nuclear receptors and coregulators. Cell.

[42] Fondell JD, Ge H, Roeder RG (1996). Ligand induction of a transcriptionally active thyroid hormone receptor coactivator complex. Proc Natl Acad Sci U S A.

[43] Singh BK, Sinha RA, Ohba K, Yen PM (2017). Role of thyroid hormone in hepatic gene regulation, chromatin remodeling, and autophagy. Mol Cell Endocrinol.

[44] Chatterjee VK, Lee JK, Rentoumis A, Jameson JL (1989). Negative regulation of the thyroid-stimulating hormone alpha gene by thyroid hormone: receptor interaction adjacent to the TATA box. Proc Natl Acad Sci U S A.

[45] Tagami T, Madison LD, Nagaya T, Jameson JL (1997). Nuclear receptor corepressors activate rather than suppress basal transcription of genes that are negatively regulated by thyroid hormone. Mol Cell Biol.

[46] Wang D, Xia X, Liu Y, Oetting A, Walker RL, Zhu Y (2009). Negative regulation of TSHalpha target gene by thyroid hormone involves histone acetylation and corepressor complex dissociation. Mol Endocrinol.

[47] Song Y, Shan S, Zhang Y, Liu W, Ding W, Ren W (2012). Ligand-dependent corepressor acts as a novel corepressor of thyroid hormone receptor and represses hepatic lipogenesis in mice. J Hepatol.

[48] Tansey WP, Catanzaro DF (1991). Sp1 and thyroid hormone receptor differentially activate expression of human growth hormone and chorionic somatomammotropin genes. J Biol Chem.

[49] Shih A, Lin HY, Davis FB, Davis PJ (2001). Thyroid hormone promotes serine phosphorylation of p53 by mitogen-activated protein kinase. Biochemistry.

[50] Kakizawa T, Miyamoto T, Ichikawa K, Takeda T, Suzuki S, Mori J (2001). Silencing mediator for retinoid and thyroid hormone receptors interacts with octamer transcription factor-1 and acts as a transcriptional repressor. J Biol Chem.

[51] Palomino T, Sanchez-Pacheco A, Pena P, Aranda A (1998). A direct protein-protein interaction is involved in the cooperation between thyroid hormone and retinoic acid receptors and the transcription factor GHF-1. FASEB J.

[52] Sanchez-Pacheco A, Pena P, Palomino T, Guell A, Castrillo JL, Aranda A (1998). The transcription factor GHF-1, but not the splice variant GHF-2, cooperates with thyroid hormone and retinoic acid receptors to stimulate rat growth hormone gene expression. FEBS Lett.

[53] Lutz M, Burke LJ, LeFevre P, Myers FA, Thorne AW, Crane-Robinson C (2003). Thyroid hormone-regulated enhancer blocking: cooperation of CTCF and thyroid hormone receptor. EMBO J.

[54] Weth O, Weth C, Bartkuhn M, Leers J, Uhle F, Renkawitz R (2010). Modular insulators: genome wide search for composite CTCF/thyroid hormone receptor binding sites. PLoS One.

[55] Davis FB, Tang HY, Shih A, Keating T, Lansing L, Hercbergs A (2006). Acting via a cell surface receptor, thyroid hormone is a growth factor for glioma cells. Cancer Res.

[56] Bergh JJ, Lin HY, Lansing L, Mohamed SN, Davis FB, Mousa S (2005). Integrin alphaVbeta3 contains a cell surface receptor site for thyroid hormone that is linked to activation of mitogen-activated protein kinase and induction of angiogenesis. Endocrinology.

[57] Cody V, Davis PJ, Davis FB (2007). Molecular modeling of the thyroid hormone interactions with alpha v beta 3 integrin. Steroids.

[58] Davis PJ, Goglia F, Leonard JL (2016). Nongenomic actions of thyroid hormone. Nat Rev Endocrinol.

[59] Cao X, Kambe F, Yamauchi M, Seo H (2009). Thyroid-hormone-dependent activation of the phosphoinositide 3-kinase/Akt cascade requires Src and enhances neuronal survival. Biochem J.

[60] Davis PJ, Davis FB (1996). Nongenomic actions of thyroid hormone. Thyroid.

[61] Cao HJ, Lin HY, Luidens MK, Davis FB, Davis PJ (2009). Cytoplasm-to-nucleus shuttling of thyroid hormone receptor-beta1 (Trbeta1) is directed from a plasma membrane integrin receptor by thyroid hormone. Endocr Res.

[62] Tang HY, Lin HY, Zhang S, Davis FB, Davis PJ (2004). Thyroid hormone causes mitogen-activated protein kinase-dependent phosphorylation of the nuclear estrogen receptor. Endocrinology.

[63] Baumann CT, Maruvada P, Hager GL, Yen PM (2001). Nuclear cytoplasmic shuttling by thyroid hormone receptors. multiple protein interactions are required for nuclear retention. J Biol Chem.

[64] Lin HY, Shih A, Davis FB, Davis PJ (1999). Thyroid hormone promotes the phosphorylation of STAT3 and potentiates the action of epidermal growth factor in cultured cells. Biochem J.

[65] Chen Y, Chen PL, Chen CF, Sharp ZD, Lee WH (1999). Thyroid hormone, T3-dependent phosphorylation and translocation of Trip230 from the Golgi complex to the nucleus. Proc Natl Acad Sci U S A.

[66] Vasudevan N, Ogawa S, Pfaff D (2002). Estrogen and thyroid hormone receptor interactions: physiological flexibility by molecular specificity. Physiol Rev.

[67] Meng R, Tang HY, Westfall J, London D, Cao JH, Mousa SA (2011). Crosstalk between integrin alphavbeta3 and estrogen receptor-alpha is involved in thyroid hormone-induced proliferation in human lung carcinoma cells. PLoS One.

[68] Hiroi Y, Kim HH, Ying H, Furuya F, Huang Z, Simoncini T (2006). Rapid nongenomic actions of thyroid hormone. Proc Natl Acad Sci U S A.

[69] Moeller LC, Cao X, Dumitrescu AM, Seo H, Refetoff S (2006). Thyroid hormone mediated changes in gene expression can be initiated by cytosolic action of the thyroid hormone receptor beta through the phosphatidylinositol 3-kinase pathway. Nucl Recept Signal.

[70] Cao X, Kambe F, Moeller LC, Refetoff S, Seo H (2005). Thyroid hormone induces rapid activation of Akt/protein kinase B-mammalian target of rapamycin-p70S6K cascade through phosphatidylinositol 3-kinase in human fibroblasts. Mol Endocrinol.

[71] Moeller LC, Dumitrescu AM, Refetoff S (2005). Cytosolic action of thyroid hormone leads to induction of hypoxia-inducible factor-1alpha and glycolytic genes. Mol Endocrinol.

[72] Storey NM, Gentile S, Ullah H, Russo A, Muessel M, Erxleben C (2006). Rapid signaling at the plasma membrane by a nuclear receptor for thyroid hormone. Proc Natl Acad Sci U S A.

[73] Lei J, Nowbar S, Mariash CN, Ingbar DH (2003). Thyroid hormone stimulates Na-K-ATPase activity and its plasma membrane insertion in rat alveolar epithelial cells. Am J Physiol Lung Cell Mol Physiol.

[74] Lei J, Mariash CN, Bhargava M, Wattenberg EV, Ingbar DH (2008). T3 increases Na-K-ATPase activity via a MAPK/ERK1/2-dependent pathway in rat adult alveolar epithelial cells. Am J Physiol Lung Cell Mol Physiol.

[75] Guigon CJ, Kim DW, Willingham MC, Cheng SY (2011). Mutation of thyroid hormone receptor-beta in mice predisposes to the development of mammary tumors. Oncogene.

[76] Guigon CJ, Cheng SY (2009). Novel oncogenic actions of TRbeta mutants in tumorigenesis. IUBMB Life.

[77] Guigon CJ, Zhao L, Lu C, Willingham MC, Cheng SY (2008). Regulation of beta-catenin by a novel nongenomic action of thyroid hormone beta receptor. Mol Cell Biol.

[78] Yorimitsu T, Klionsky DJ (2005). Autophagy: molecular machinery for self-eating. Cell Death Differ.

[79] Mizushima N, Levine B, Cuervo AM, Klionsky DJ (2008). Autophagy fights disease through cellular self-digestion. Nature.

[80] Ciechanover A, Orian A, Schwartz AL (2000). Ubiquitin-mediated proteolysis: biological regulation via destruction. Bioessays.

[81] Nedelsky NB, Todd PK, Taylor JP (2008). Autophagy and the ubiquitin-proteasome system: collaborators in neuroprotection. Biochim Biophys Acta.

[82] Khaminets A, Behl C, Dikic I (2016). Ubiquitin-Dependent And Independent Signals In Selective Autophagy. Trends Cell Biol.

[83] Rogov V, Dotsch V, Johansen T, Kirkin V (2014). Interactions between autophagy receptors and ubiquitin-like proteins form the molecular basis for selective autophagy. Mol Cell.

[84] Fujita N, Morita E, Itoh T, Tanaka A, Nakaoka M, Osada Y (2013). Recruitment of the autophagic machinery to endosomes during infection is mediated by ubiquitin. J Cell Biol.

[85] Lazarou M, Sliter DA, Kane LA, Sarraf SA, Wang C, Burman JL (2015). The ubiquitin kinase PINK1 recruits autophagy receptors to induce mitophagy. Nature.

[86] Ueno T, Komatsu M (2017). Autophagy in the liver: functions in health and disease. Nat Rev Gastroenterol Hepatol.

[87] Kim IH, Kisseleva T, Brenner DA (2015). Aging and liver disease. Curr Opin Gastroenterol.

[88] Vittorini S, Paradiso C, Donati A, Cavallini G, Masini M, Gori Z (1999). The age-related accumulation of protein carbonyl in rat liver correlates with the age-related decline in liver proteolytic activities. J Gerontol A Biol Sci Med Sci.

[89] Cavallini G, Donati A, Gori Z, Pollera M, Bergamini E (2001). The protection of rat liver autophagic proteolysis from the age-related decline co-varies with the duration of anti-ageing food restriction. Exp Gerontol.

[90] Donati A, Cavallini G, Paradiso C, Vittorini S, Pollera M, Gori Z (2001). Age-related changes in the regulation of autophagic proteolysis in rat isolated hepatocytes. J Gerontol A Biol Sci Med Sci.

[91] Uddin MN, Nishio N, Ito S, Suzuki H, Isobe K (2012). Autophagic activity in thymus and liver during aging. Age (Dordr).

[92] Farrell GC, Larter CZ (2006). Nonalcoholic fatty liver disease: from steatosis to cirrhosis. Hepatology.

[93] Das K, Kar P (2005). Non-alcoholic steatohepatitis. J Assoc Physicians India.

[94] Ota T, Takamura T, Kurita S, Matsuzawa N, Kita Y, Uno M (2007). Insulin resistance accelerates a dietary rat model of nonalcoholic steatohepatitis. Gastroenterology.

[95] Brenner C, Galluzzi L, Kepp O, Kroemer G (2013). Decoding cell death signals in liver inflammation. J Hepatol.

[96] Yoon HJ, Cha BS (2014). Pathogenesis and therapeutic approaches for non-alcoholic fatty liver disease. World J Hepatol.

[97] Singh R, Kaushik S, Wang Y, Xiang Y, Novak I, Komatsu M (2009). Autophagy regulates lipid metabolism. Nature.

[98] Fukuo Y, Yamashina S, Sonoue H, Arakawa A, Nakadera E, Aoyama T (2014). Abnormality of autophagic function and cathepsin expression in the liver from patients with non-alcoholic fatty liver disease. Hepatol Res.

[99] Park HW, Park H, Semple IA, Jang I, Ro SH, Kim M (2014). Pharmacological correction of obesity-induced autophagy arrest using calcium channel blockers. Nat Commun.

[100] Lin CW, Zhang H, Li M, Xiong X, Chen X, Dong XC (2013). Pharmacological promotion of autophagy alleviates steatosis and injury in alcoholic and non-alcoholic fatty liver conditions in mice. J Hepatol.

[101] Sinha RA, Farah BL, Singh BK, Siddique MM, Li Y, Wu Y (2014). Caffeine stimulates hepatic lipid metabolism by the autophagy-lysosomal pathway in mice. Hepatology.

[102] Sun L, Zhang S, Yu C, Pan Z, Liu Y, Zhao J (2015). Hydrogen sulfide reduces serum triglyceride by activating liver autophagy via the AMPK-mTOR pathway. Am J Physiol Endocrinol Metab.

[103] Friedman SL (2008). Mechanisms of hepatic fibrogenesis. Gastroenterology.

[104] Hernandez-Gea V, Ghiassi-Nejad Z, Rozenfeld R, Gordon R, Fiel MI, Yue Z (2012). Autophagy releases lipid that promotes fibrogenesis by activated hepatic stellate cells in mice and in human tissues. Gastroenterology.

[105] Lavallard VJ, Gual P (2014). Autophagy and non-alcoholic fatty liver disease. Biomed Res Int.

[106] Hayes JD, McMahon M (2009). NRF2 and KEAP1 mutations: permanent activation of an adaptive response in cancer. Trends Biochem Sci.

[107] Taguchi K, Motohashi H, Yamamoto M (2011). Molecular mechanisms of the Keap1-Nrf2 pathway in stress response and cancer evolution. Genes Cells.

[108] White E (2015). The role for autophagy in cancer. J Clin Invest.

[109] Rao S, Tortola L, Perlot T, Wirnsberger G, Novatchkova M, Nitsch R (2014). A dual role for autophagy in a murine model of lung cancer. Nat Commun.

[110] Guo JY, Karsli-Uzunbas G, Mathew R, Aisner SC, Kamphorst JJ, Strohecker AM (2013). Autophagy suppresses progression of K-ras-induced lung tumors to oncocytomas and maintains lipid homeostasis. Genes Dev.

[111] Jiang P, Mizushima N (2014). Autophagy and human diseases. Cell Res.

[112] Zhi X, Zhong Q (2015). Autophagy in cancer. F1000Prime Rep.

[113] Rosenfeldt MT, O'Prey J, Morton JP, Nixon C, MacKay G, Mrowinska A (2013). p53 status determines the role of autophagy in pancreatic tumour development. Nature..

[114] Takamura A, Komatsu M, Hara T, Sakamoto A, Kishi C, Waguri S (2011). Autophagy-deficient mice develop multiple liver tumors. Genes Dev.

[115] Inami Y, Waguri S, Sakamoto A, Kouno T, Nakada K, Hino O (2011). Persistent activation of Nrf2 through p62 in hepatocellular carcinoma cells. J Cell Biol.

[116] Qu X, Yu J, Bhagat G, Furuya N, Hibshoosh H, Troxel A (2003). Promotion of tumorigenesis by heterozygous disruption of the beclin 1 autophagy gene. J Clin Invest.

[117] Ni HM, Woolbright BL, Williams J, Copple B, Cui W, Luyendyk JP (2014). Nrf2 promotes the development of fibrosis and tumorigenesis in mice with defective hepatic autophagy. J Hepatol.

[118] Lozy F, Karantza V (2012). Autophagy and cancer cell metabolism. Semin Cell Dev Biol.

[119] Yue Z, Jin S, Yang C, Levine AJ, Heintz N (2003). Beclin 1, an autophagy gene essential for early embryonic development, is a haploinsufficient tumor suppressor. Proc Natl Acad Sci U S A.

[120] Ding ZB, Shi YH, Zhou J, Qiu SJ, Xu Y, Dai Z (2008). Association of autophagy defect with a malignant phenotype and poor prognosis of hepatocellular carcinoma. Cancer Res.

[121] Kanzawa T, Kondo Y, Ito H, Kondo S, Germano I (2003). Induction of autophagic cell death in malignant glioma cells by arsenic trioxide. Cancer Res.

[122] Kim EH, Sohn S, Kwon HJ, Kim SU, Kim MJ, Lee SJ (2007). Sodium selenite induces superoxide-mediated mitochondrial damage and subsequent autophagic cell death in malignant glioma cells. Cancer Res.

[123] Dupere-Richer D, Kinal M, Menasche V, Nielsen TH, Del Rincon S, Pettersson F (2013). Vorinostat-induced autophagy switches from a death-promoting to a cytoprotective signal to drive acquired resistance. Cell Death Dis.

[124] Yap TA, Garrett MD, Walton MI, Raynaud F, de Bono JS, Workman P (2008). Targeting the PI3K-AKT-mTOR pathway: progress, pitfalls, and promises. Curr Opin Pharmacol.

[125] Sieghart W, Fuereder T, Schmid K, Cejka D, Werzowa J, Wrba F (2007). Mammalian target of rapamycin pathway activity in hepatocellular carcinomas of patients undergoing liver transplantation. Transplantation.

[126] Villanueva A, Chiang DY, Newell P, Peix J, Thung S, Alsinet C (2008). Pivotal role of mTOR signaling in hepatocellular carcinoma. Gastroenterology.

[127] Decaens T, Luciani A, Itti E, Hulin A, Roudot-Thoraval F, Laurent A (2012). Phase II study of sirolimus in treatment-naive patients with advanced hepatocellular carcinoma. Dig Liver Dis.

[128] Toso C, Merani S, Bigam DL, Shapiro AM, Kneteman NM (2010). Sirolimus-based immunosuppression is associated with increased survival after liver transplantation for hepatocellular carcinoma. Hepatology.

[129] Huynh H, Chow KH, Soo KC, Toh HC, Choo SP, Foo KF (2009). RAD001 (everolimus) inhibits tumour growth in xenograft models of human hepatocellular carcinoma. J Cell Mol Med.

[130] Zhu AX, Kudo M, Assenat E, Cattan S, Kang YK, Lim HY (2014). Effect of everolimus on survival in advanced hepatocellular carcinoma after failure of sorafenib: the EVOLVE-1 randomized clinical trial. Jama.

[131] Thomas HE, Mercer CA, Carnevalli LS, Park J, Andersen JB, Conner EA (2012). mTOR inhibitors synergize on regression, reversal of gene expression, and autophagy in hepatocellular carcinoma. Sci Transl Med.

[132] Egan DF, Chun MG, Vamos M, Zou H, Rong J, Miller CJ (2015). Small Molecule Inhibition of the Autophagy Kinase ULK1 and Identification of ULK1 Substrates. Mol Cell.

[133] Gauthier A, Ho M (2013). Role of sorafenib in the treatment of advanced hepatocellular carcinoma: An update. Hepatol Res.

[134] DeMartino GN, Goldberg AL (1978). Thyroid hormones control lysosomal enzyme activities in liver and skeletal muscle. Proc Natl Acad Sci U S A.

[135] Webb AE, Brunet A (2014). FOXO transcription factors: key regulators of cellular quality control. Trends in biochemical sciences.

[136] Singh BK, Sinha RA, Zhou J, Tripathi M, Ohba K, Wang ME (2016). Hepatic FOXO1 Target Genes Are Co-regulated by Thyroid Hormone via RICTOR Protein Deacetylation and MTORC2-AKT Protein Inhibition. J Biol Chem.

[137] Singh BK, Sinha RA, Zhou J, Xie SY, You SH, Gauthier K (2013). FoxO1 deacetylation regulates thyroid hormone-induced transcription of key hepatic gluconeogenic genes. J Biol Chem.

[138] Weitzel JM, Iwen KA (2011). Coordination of mitochondrial biogenesis by thyroid hormone. Mol Cell Endocrinol.

[139] Matsumoto G, Wada K, Okuno M, Kurosawa M, Nukina N (2011). Serine 403 phosphorylation of p62/SQSTM1 regulates selective autophagic clearance of ubiquitinated proteins. Mol Cell.

[140] Lim J, Lachenmayer ML, Wu S, Liu W, Kundu M, Wang R (2015). Proteotoxic stress induces phosphorylation of p62/SQSTM1 by ULK1 to regulate selective autophagic clearance of protein aggregates. PLoS Genet.

[141] Cingolani F, Czaja MJ (2016). Regulation and Functions of Autophagic Lipolysis. Trends Endocrinol Metab.

[142] Cioffi F, Senese R, Lanni A, Goglia F (2013). Thyroid hormones and mitochondria: with a brief look at derivatives and analogues. Mol Cell Endocrinol.

[143] Koyano F, Okatsu K, Kosako H, Tamura Y, Go E, Kimura M (2014). Ubiquitin is phosphorylated by PINK1 to activate parkin. Nature.

[144] Kane LA, Lazarou M, Fogel AI, Li Y, Yamano K, Sarraf SA (2014). PINK1 phosphorylates ubiquitin to activate Parkin E3 ubiquitin ligase activity. J Cell Biol.

[145] Baxter JD, Webb P (2009). Thyroid hormone mimetics: potential applications in atherosclerosis, obesity and type 2 diabetes. Nat Rev Drug Discov.

[146] Baxter JD, Dillmann WH, West BL, Huber R, Furlow JD, Fletterick RJ (2001). Selective modulation of thyroid hormone receptor action. J Steroid Biochem Mol Biol.

[147] Webb P (2004). Selective activators of thyroid hormone receptors. Expert Opin Investig Drugs.

[148] Moreno M, de Lange P, Lombardi A, Silvestri E, Lanni A, Goglia F (2008). Metabolic effects of thyroid hormone derivatives. Thyroid.

[149] Brenta G, Danzi S, Klein I (2007). Potential therapeutic applications of thyroid hormone analogs. Nat Clin Pract Endocrinol Metab.

[150] Grover GJ, Egan DM, Sleph PG, Beehler BC, Chiellini G, Nguyen NH (2004). Effects of the thyroid hormone receptor agonist GC-1 on metabolic rate and cholesterol in rats and primates: selective actions relative to 3,5,3'-triiodo-L-thyronine. Endocrinology.

[151] Trost SU, Swanson E, Gloss B, Wang-Iverson DB, Zhang H, Volodarsky T (2000). The thyroid hormone receptor-beta-selective agonist GC-1 differentially affects plasma lipids and cardiac activity. Endocrinology.

[152] Ledda-Columbano GM, Perra A, Piga R, Pibiri M, Loi R, Shinozuka H (1999). Cell proliferation induced by 3,3',5-triiodo-L-thyronine is associated with a reduction in the number of preneoplastic hepatic lesions. Carcinogenesis.

[153] Puliga E, Min Q, Tao J, Zhang R, Pradhan-Sundd T, Poddar M (2017). Thyroid Hormone Receptor-beta Agonist GC-1 Inhibits Met-beta-Catenin-Driven Hepatocellular Cancer. Am J Pathol.

[154] Erion MD, Cable EE, Ito BR, Jiang H, Fujitaki JM, Finn PD (2007). Targeting thyroid hormone receptor-beta agonists to the liver reduces cholesterol and triglycerides and improves the therapeutic index. Proc Natl Acad Sci U S A.

[155] Berkenstam A, Kristensen J, Mellstrom K, Carlsson B, Malm J, Rehnmark S (2008). The thyroid hormone mimetic compound KB2115 lowers plasma LDL cholesterol and stimulates bile acid synthesis without cardiac effects in humans. Proc Natl Acad Sci U S A.

[156] Martagon AJ, Lin JZ, Cimini SL, Webb P, Phillips KJ (2015). The amelioration of hepatic steatosis by thyroid hormone receptor agonists is insufficient to restore insulin sensitivity in ob/ob mice. PLoS One.

[157] Ladenson PW, Kristensen JD, Ridgway EC, Olsson AG, Carlsson B, Klein I (2010). Use of the thyroid hormone analogue eprotirome in statin-treated dyslipidemia. N Engl J Med.

[158] Szydlowska M, Pibiri M, Perra A, Puliga E, Mattu S, Ledda-Columbano GM (2017). The Thyromimetic KB2115 (Eprotirome) Induces Rat Hepatocyte Proliferation. Gene Expr.

[159] Sjouke B, Langslet G, Ceska R, Nicholls SJ, Nissen SE, Ohlander M (2014). Eprotirome in patients with familial hypercholesterolaemia (the AKKA trial): a randomised, double-blind, placebo-controlled phase 3 study. Lancet Diabetes Endocrinol.

[160] Kelly MJ, Pietranico-Cole S, Larigan JD, Haynes NE, Reynolds CH, Scott N (2014). Discovery of 2-[3,5-dichloro-4-(5-isopropyl-6-oxo-1,6-dihydropyridazin-3-yloxy)phenyl]-3,5-dio xo-2,3,4,5-tetrahydro[1,2,4]triazine-6-carbonitrile (MGL-3196), a Highly Selective Thyroid Hormone Receptor beta agonist in clinical trials for the treatment of dyslipidemia. J Med Chem.

[161] Taub R, Chiang E, Chabot-Blanchet M, Kelly MJ, Reeves RA, Guertin MC (2013). Lipid lowering in healthy volunteers treated with multiple doses of MGL-3196, a liver-targeted thyroid hormone receptor-beta agonist. Atherosclerosis.

[162] Scherz-Shouval R, Elazar Z (2011). Regulation of autophagy by ROS: physiology and pathology. Trends Biochem Sci.

[163] Zatloukal K, Stumptner C, Fuchsbichler A, Heid H, Schnoelzer M, Kenner L (2002). p62 Is a common component of cytoplasmic inclusions in protein aggregation diseases. Am J Pathol.

[164] Zatloukal K, French SW, Stumptner C, Strnad P, Harada M, Toivola DM (2007). From Mallory to Mallory-Denk bodies: what, how and why?. Exp Cell Res.

[165] Kremsdorf D, Soussan P, Paterlini-Brechot P, Brechot C (2006). Hepatitis B virus-related hepatocellular carcinoma: paradigms for viral-related human carcinogenesis. Oncogene.

[166] Xu L, Ma H, Miao M, Li Y (2012). Impact of subclinical hypothyroidism on the development of non-alcoholic fatty liver disease: a prospective case-control study. J Hepatol.

[167] Liu Y, Wang W, Yu X, Qi X (2018). Thyroid Function and Risk of Non-Alcoholic Fatty Liver Disease in Euthyroid Subjects. Ann Hepatol.

[168] Guo Z, Li M, Han B, Qi X (2018). Association of non-alcoholic fatty liver disease with thyroid function: A systematic review and meta-analysis. Dig Liver Dis.

[169] Mandato C, D'Acunzo I, Vajro P (2018). Thyroid dysfunction and its role as a risk factor for non-alcoholic fatty liver disease: What's new. Dig Liver Dis.

[170] Liu L, Yu Y, Zhao M, Zheng D, Zhang X, Guan Q (2017). Benefits of Levothyroxine Replacement Therapy on Nonalcoholic Fatty Liver Disease in Subclinical Hypothyroidism Patients. Int J Endocrinol.

[171] Wu SM, Cheng WL, Liao CJ, Chi HC, Lin YH, Tseng YH (2015). Negative modulation of the epigenetic regulator, UHRF1, by thyroid hormone receptors suppresses liver cancer cell growth. Int J Cancer.

[172] Tseng YH, Huang YH, Lin TK, Wu SM, Chi HC, Tsai CY (2016). Thyroid hormone suppresses expression of stathmin and associated tumor growth in hepatocellular carcinoma. Sci Rep.

[173] Huang PS, Lin YH, Chi HC, Chen PY, Huang YH, Yeh CT (2017). Thyroid hormone inhibits growth of hepatoma cells through induction of miR-214. Sci Rep.

[174] Lin YH, Wu MH, Huang YH, Yeh CT, Chi HC, Tsai CY (2018). Thyroid hormone negatively regulates tumorigenesis through suppression of BC200. Endocr Relat Cancer.

[175] Chi HC, Chen SL, Liao CJ, Liao CH, Tsai MM, Lin YH (2012). Thyroid hormone receptors promote metastasis of human hepatoma cells via regulation of TRAIL. Cell Death Differ.

[176] Chi HC, Chen SL, Cheng YH, Lin TK, Tsai CY, Tsai MM (2016). Chemotherapy resistance and metastasis-promoting effects of thyroid hormone in hepatocarcinoma cells are mediated by suppression of FoxO1 and Bim pathway. Cell Death Dis.

[177] Lin YH, Wu MH, Liao CJ, Huang YH, Chi HC, Wu SM (2015). Repression of microRNA-130b by thyroid hormone enhances cell motility. J Hepatol.

[178] Chen CY, Chung IH, Tsai MM, Tseng YH, Chi HC, Tsai CY (2014). Thyroid hormone enhanced human hepatoma cell motility involves brain-specific serine protease 4 activation via ERK signaling. Mol Cancer.

[179] Huang YH, Lin YH, Chi HC, Liao CH, Liao CJ, Wu SM (2013). Thyroid hormone regulation of miR-21 enhances migration and invasion of hepatoma. Cancer Res.

[180] Chung IH, Chen CY, Lin YH, Chi HC, Huang YH, Tai PJ (2015). Thyroid hormone-mediated regulation of lipocalin 2 through the Met/FAK pathway in liver cancer. Oncotarget.

[181] White E, DiPaola RS (2009). The double-edged sword of autophagy modulation in cancer. Clin Cancer Res.

[182] Tian Y, Kuo CF, Sir D, Wang L, Govindarajan S, Petrovic LM (2015). Autophagy inhibits oxidative stress and tumor suppressors to exert its dual effect on hepatocarcinogenesis. Cell Death Differ.

[183] Sui X, Chen R, Wang Z, Huang Z, Kong N, Zhang M (2013). Autophagy and chemotherapy resistance: a promising therapeutic target for cancer treatment. Cell Death Dis.

[184] Sheng J, Qin H, Zhang K, Li B, Zhang X (2018). Targeting autophagy in chemotherapy-resistant of hepatocellular carcinoma. Am J Cancer Res.

